# Promising Terpenes as Natural Antagonists of Cancer: An In-Silico Approach

**DOI:** 10.3390/molecules25010155

**Published:** 2019-12-30

**Authors:** Ziyad Tariq Muhseen, Guanglin Li

**Affiliations:** 1 Key Laboratory of Ministry of Education for Medicinal Plant Resource and Natural Pharmaceutical Chemistry, Shaanxi Normal University, Xi’an 710062, China; ziyad.tariq82@gmail.com; 2 School of Life Sciences, Shaanxi Normal University, Xi’an 710062, China

**Keywords:** cancer, p53-MDM2, natural compounds, terpenes, computational analyses

## Abstract

Overexpression of murine double minute 2 (MDM2) results in the inactivation of p53 and causes cancer which is a leading cause of death in recent era. In recent decades, much attention has been paid to discover potential inhibitors against MDM2 in order to cure cancer. Outcomes from studies proposes that the MDM2 is a hot target to screen potent antagonists. Thus, this study aims at discovering natural compounds using several computational approaches to inhibit the MDM2 and to eliminate p53-MDM2 interaction, which would result in the reactivation of p53 activity. A library of 500 terpenes was prepared and several virtual screening approaches were employed to find out the best hits which could serve as p53-MDM2 antagonists. On the basis of the designed protocol, three terpenes were selected. In the present study, for the stability and validation of selected three protein-ligand complexes 20 ns molecular dynamics simulations and principal component analyses (PCA) were performed. Results found that the selected terpenes hits (3-*trans*-*p*-coumaroyl maslinic acid, Silvestrol and Betulonic acid) are potential inhibitors of p53–MDM2 interaction and could serve as potent antagonists.

## 1. Introduction

Cancer is a leading cause of death around the globe in which 56% of total affected population of the world lies in Asia, that is 44% of total global burden with 51% death toll all around the globe [1,2,3]. During cancer development normal cells are damaged and turned into cancerous cells through various factors such as cellular signaling stress, DNA damage, disturbance in intercellular and intracellular responses, loss of response to minor nutrients and replacement with carcinogenic material in unhealthy conditions [4]. Nature created mechanisms to overcome and nullify the effects of such conditions in the body. Several beneficial physio-pathological factors exist in the body for the development and maintenance of cellular communication among the cells [5]. The guardian of genome, p53, is a transcription factor that controls the cellular mechanisms of several pathways. p53 is reported to be involved in quashing tumors by regulating the cell cycle, repairing DNA and performing apoptosis [6,7]. Under conditions of stress, genetic variation and DNA damage, the cells with genetic lesions are prone to undergo uncontrolled proliferation, which can lead to the development of an unnecessary mass. Under such conditions, elevated levels of p53 play a vital role in the arrest of the cell cycle at G1-S and G2-M barriers, induce DNA repair, and ultimately save the cell from carcinogenesis [8,9]. Unfortunately, the improper functioning, alteration/mutations and inactivation of p53 gene are the major reasons for the development of cancer [10,11]. It is estimated that 50% of cancers are the outcome of alteration/mutations in the p53 gene [12], whereas in the remaining 50% of tumors the p53 wild type is retained but with inhibited cell cycle arrest [5,13]. The inhibition is caused by another co-protein known as murine double minute 2 (MDM2), which is E3 ubiquitin ligase and can regulate p53 in a negative manner. In a normal cell, MDM2 and p53 are regulated in a well-organized manner, forming an autoregulatory loop as p53 needs to be inactivated under tranquil conditions [5,14]. Structurally MDM2 comprises a highly conserved functional domain starting from the N terminus; there is a p53 binding domain, exporting signals and nuclear localization after that zinc finger domain, acidic domain and RING finger domain [15,16,17]. The p53 binding domain of MDM2 binds to the p53 transactivation domain and restrains the transcription of p53, thus transmitting it to the cytoplasm and degrading it [18]. In fact, p53 exists in unphosphorylated form in the normal cell environment; it can bind to the promoter of MDM2, and induce expression of MDM2. During DNA damage, p53 is activated by phosphorylation and does not allow MDM2 to bind to p53 and hinder degradation. After repairing the DNA damage, p53 dephosphorylated with the formation of the MDM2-p53 complex, thus controlling its degradation by means of E3 ubiquitin ligase activity of MDM2 [5,19]. The amplification and overexpression of MDM2 with a single-nucleotide polymorphism at nucleotide 309 of the promoter leads to almost 7% of malignancies in humans, including cancers of the breast, lungs, brain, ovary, cervix, colon, bone, prostate, and renal along with soft tissue sarcomas [5,20,21].

The natural compounds, mainly phytochemicals (secondary metabolites), present in many medicinal plants have significant anticancer potential because of their multiple medicinal and nutraceutical properties [5,22,23]. Currently, the use of plant-based bioactive extracts and phytomedicines is gaining recognition as these have structural diversity, negligible side effects, and bioavailability, as well as exhibit multiple target activities [24]. Plants are composed up of numerous bioactive compounds such as peptides, alkaloids, flavonoids, and terpenes [25,26]. Among these, terpenes be owned by the largest category of secondary metabolites and fundamentally made up five carbon isoprene units that are linked with each other through thousand ways. Terpenes are simple hydrocarbons while terpenoids are modified category of terpenes, which have many functional groups, in which oxidative methyl moves or replaces in different positions [27]. Terpenoids are classified into monoterpenes, diterpenes, sesterpenes, and triterpenes subjected to the units of carbon. With the change of terpenoids structure, most terpenoids have biological activity and are used to treat many diseases in the world. Many terpenoids have inhibitory effects on various human tumor cells and are used as antitumor drugs such as Taxol and its derivatives. Since, owing to the fragrance of terpenes, many flavors and fragrances are made up of terpenes. Terpenes and their derivatives have antimalarial effects, such as artemisinin and other compounds. At the same time, terpenoids have an important role in food, medicine, cosmetics, hormones, vitamins and other fields [28].

With the emergence of new and efficient computer-aided drug-designing tools the discovery of therapeutic agents has become a cost-effective, reliable and efficient process. In this regard, virtual screening, ligand docking with a known three-dimensional (3D) protein structure and molecular dynamics and simulation (MDS) have been proven to be important methods in discovering and designing the novel anti-cancerous compounds [5,22,29]. Several success stories have been reported, in which researchers successfully discover potential inhibitors of target proteins using computational approaches [5,22,29,30]. In this proposed study, several terpenes from various plants will be screened against MDM2 to stop p53-MDM2 interactions using in-silico approach. The prime objective of the research is to target the hotspot hydrophobic residues that are involved in binding with p53. We will screen out terpenes which can act as antagonists and bind to the hydrophobic cleft of MDM2 to block p53 binding, which results in curing p53-MDM2-related malignancies. Complete protocol flowchart of present study is presented in Supplementary Figure S1. The results of this study will reinforce the efficacy of anticancer terpenes and give a thrust to its development as a drug compound.

## 2. Results and Discussion

Cancer is a serious health issue and responsible for one in eight deaths across the globe [5]. The overexpression, misregulation of transcription and translation of MDM2 may result in the development of malignancies. This overexpression is a consequence of a single-nucleotide polymorphism at the 309 position in the promoter section of MDM2 [14]. The binding cleft of MDM2 with p53 has an interaction capability with the 109-amino-acid-long NH2 terminal domain consisting of four α helices and six β sheets where amphipathic helix of transactivation domain comprised eight residues (reside 18–26) of p53 contact forming intermolecular hydrogen bonds [14]. This interaction enabled scientists to follow several experimental and computer-aided drug-designing approaches to develop antagonists that have the potential to inhibit the interaction between the two proteins [5,8,9,13,14]. The intensive research yielded compounds such as nutlin-3a [31], Spirooxindoles [32], benzodiazepinediones, MI-219 [33] and Epigallocatechin gallate, alvaradoin M, alvaradoin E and nordihydroguaiaretic acid [5]. Synthetic chemical compounds may possess serious bio-susceptibility and health issues. In the current investigation, natural bio-active compounds were screened to enhance the efficacy and biocompatibility and to exploit toxicity-related health problems. Bio-active compounds, as a part of natural medicinal plants, have negligible side effects and have been used for decades in traditional medicines. The potency of plant extracts in cancer control has been explored over the decades [22]. The current study focused on discovering novel terpenes as antagonists of p53-MDM2 interaction to reactivate p53 running using several virtual screening approaches coupled with MDS.

### 2.1. Protocol Validation and Receptor Selection

**Figure 1:**
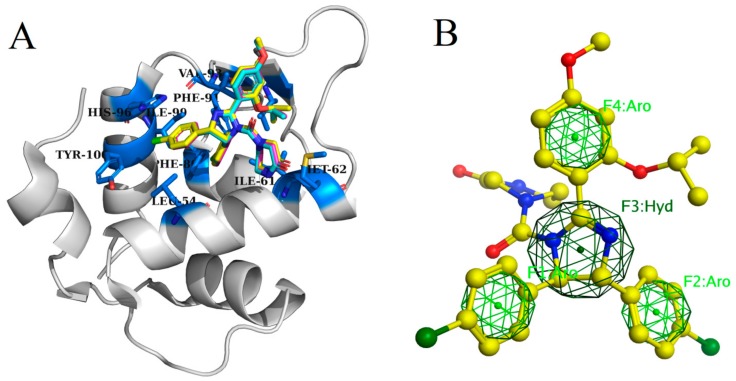
(**A**) Binding of all ligands inside the binding pocket of MDM2, (**B**) 3D representation of pharmacophore features model used to search against the terpenes library.

In order to verify the prediction of docking protocol ability, reference ligand was redocked to maintain the rigidity and flexibility of protein active sites to MDM2 (PDB ID: 4HG7). The receptor was selected based on their capability for forecasting suitable binding mode. RMSD of flexibly redocked ligand and rigid body docked ligand was 1.0 Å. The results reveal that the docking protocol is effective for predicting the binding attitude. Figure 1A shows that the docked conformation of rigid body redocked ligand (magenta), flexibly redocked ligand (yellow) and the X-ray crystal structure (blue) are identical.

### 2.2. Pharmacophore Based Virtual Screening and Validation of Drug-Likeness

In this study, MOE pharmacophore constructing tool was used to construct complex pharmacophore model. Combined with the interaction, important chemical characteristics were induced, which were considered in the establishment of pharmacophore model. A noteworthy binding interaction was perceived in the protein ligand complex by MOE ligplot tool. Using the default factors of MOE, 4 key characteristics were generated in the pharmacophore model, including three aromatic (Aro) as well as one hydrogen bond (Hyd) (Figure 1B). Similarly, the generated pharmacophore system was assessed by means of test database of an already known inhibitors. Based on the characteristic complex pharmacophore model, all the inhibitors in the experimental database and their mapping modes were evaluated. The evaluation results show that most of the active compounds meet the criteria. These hits show that four features of 50 different conformations that create a map of the pharmacophore system. No inactive drug was mapped to any feature of the complex based pharmacophore system. The findings of the test database show the accuracy of the pharmacophore model. There are several reasons why such screening was adopted firstly, the validated pharmacophore model has been effectively applied for the proper identification of compounds with known anticancer potential and secondly, the significance of this technique for further evaluation is to identify new and effective generic drug programs [30,34,35]. Pharmacophore model based virtual screening was performed on prepared terpenes database containing 500 different compounds from several plants using MOE pharmacophore tool. The best hit compounds with analogous characteristics and new structural conformation were selected from the terpenese database. Based on the screening of pharmacophore model, 157 results with various structures were obtained from the terpenes database, which strengthened the four characteristic concepts of pharmacophore model. To further enrich the dataset, similarity search based on Tanimoto coefficient was also applied using nutlin-3a to search for similar compounds, i.e., functional groups with similar shapes and reference ligands. [36]. Total 90 compounds with a value of Tanimoto coefficient >0.4 were chosen from developed terpenes database for further docking studies. Next, drug capability of these 247 hit compounds (157 from pharmacophore search and 90 from tanimoto coefficient search) was tested using relaxed Lipinski’s five rule. Only 80 terpenes passed this test had been used for molecular docking.

### 2.3. Molecular Docking

The terpenes screened using the pharmacophore model, similarity and virtual search, were then docked into the hydrophobic binding pocket of MDM2 by usingof Autodock Vina. After docking, compounds were ranked based on the biding energy score. The docking score of the reference ligand (Nutlin-3a) was −12.67 kcal/mol The only compounds those have higher binding score compared to the natural substrate, can be classified as potential inhibitors [30,35]. Therefore, only those compounds which have docking score higher than reference ligand Nutlin-3a score (−12.67 kcal/mol), were further considered for second step of docking. In total, 53 compounds fulfilled this criterion. In second phase of docking, selected 53 compounds were flexibly docked into the hydrophobic binding pocket of MDM2. After the flexible docking, the docked compounds were clustered into three clusters using k-means algorithm. One representative compound from each cluster was picked for further analysis. Table 1 shows the chemical attributes and structures of the three finally screened compounds that bound deep inside the hydrophobic binding pocket and exhibited hydrophobic interaction with the active site residues; Gln 24, Leu 54, Ile 61, Met 62, Val 75, Phe 86, Phe 91, Val 93, His 96, Ile 99 and Tyr 100 (Supplementary Figure S2).

**Figure 2:**
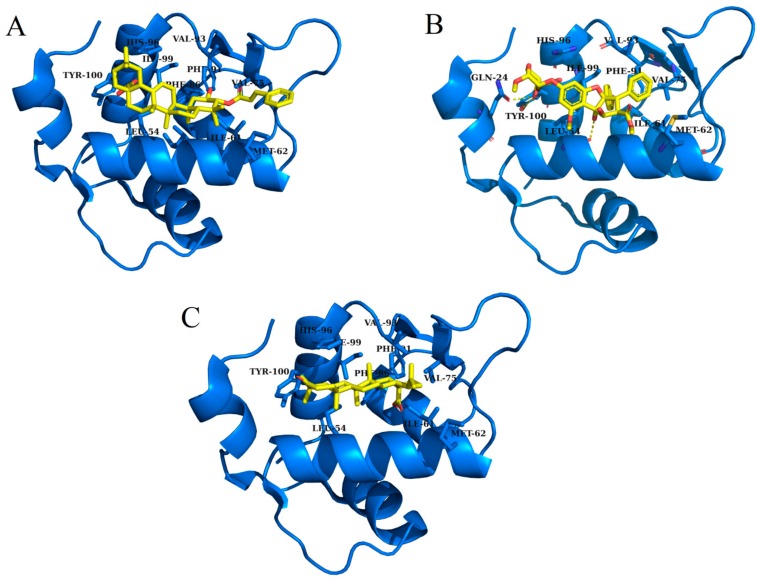
Illustration of terpenes binding inside MDM2 active site. (**A**) 3-*trans*-*p*-coumaroyl maslinic acid, (**B**) Silvestrol and (**C**) Betulonic aci.

Table 2 and Figure 2 summarize the binding site residues and binding energy score, which interact with compounds. Among all complexes, 3-*trans*-*p*-coumaroyl maslinic acid ranked at first which was forming hydrophobic interactions with Leu 54, Ile 61, Met 62, Val 75, Phe 86, Phe 91, Val 93, His 96, Ile 99 and Tyr 100, with energy score −22.60 kcal/mol. 3-*trans*-*p*-coumaroyl maslinic acid usually obtained from *Ziziphus jujuba* has significant anti-cancer activities reported [37,40]. Silvestrol ranked at second with energy score −20.75 kcal/mol while Betulonic acid ranked third with energy score −18.83 kcal/mol Silvestrol is found in *Aglaia silvestris* and has been studied extensively in several anticancer studies [38,41,42,43,44,45,46]. Betulonic acid is found in *Fructus Jujubae* and also possess anti-tumor potential [39,47]. All compounds were found to have suitable energy score and Hydrogen Bond interactions with the hydrophobic binding pocket of MDM2. The binding energy scores of finally selected terpenes were not even better than native ligand Nutlin-3a but also found higher compared with a recent study which reported 4 phytochemicals including Epigallocatechin gallate, alvaradoin M, alvaradoin E and nordihydroguaiaretic acid having binding energy scores −17.40, −14.22, −12.19 and −11.54 respectively, as natural inhibitors of p53-MDM2 interaction to reactivate p53 functioning [5]. For further exploration, the applicability and stability of these complexes were simulated by molecular dynamics simulation.

### 2.4. Molecular Dynamics Simulations

Molecular dynamics (MD) simulation is a common method to study the micro interaction between protein and ligand structure [48]. In order to further assess its dynamics and stability, all complexes were simulated by 20 ns MD followed by secondary structure and principle component analysis.

#### 2.4.1. Root Mean Square Deviations (RMSD)

**Figure 3:**
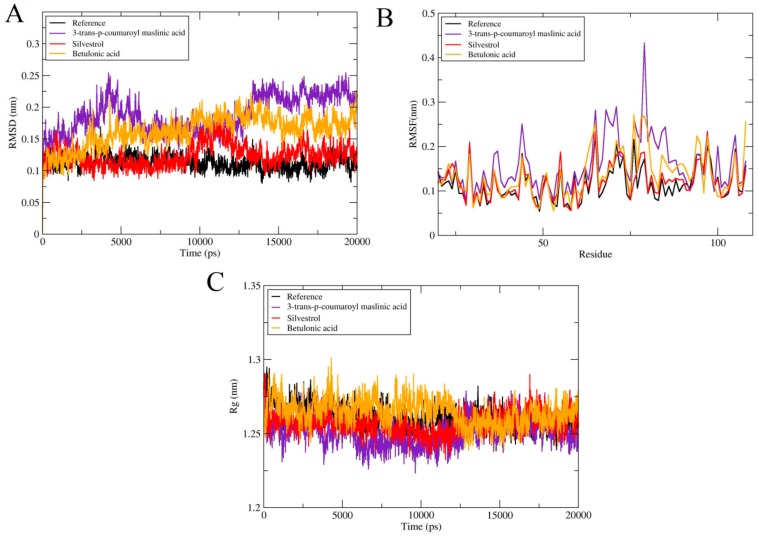
Comparison and detail representation of (**A**) RMSD: Root mean square deviations, (**B**) RMSF: Root mean square fluctuations and (**C**) Potential energy over the time of 20 ns of Reference and all terpenes complexes.

The values of RMSD of backbone atoms were computed to monitor the stability of complexes (Figure 3A). The RMSD average values for all these complexes Reference, 3-*trans*-*p*-coumaroyl maslinic acid, Silvestrol, Betulonic acid are 3 Å, 2.4 Å, 2.7 Å and 2.8 Å, respectively. All the systems except Reference varied up to 12 ns and then subsequently system is stabled until 20 ns, proposing that the system is folded to a steadier state than the initial structure. There were no significant variations noticed in all docked complexes. In case of 3-*trans*-*p*-coumaroyl maslinic acid, minute obvious changes were noticed with average RMSD 2.5 Å, but on the whole, there was no obvious fluctuation in all docked complexes.

#### 2.4.2. Root Mean Square Fluctuations (RMSF)

To further compute, the residual flexibility root means square fluctuations (RMSF) over 20 ns time were computed. The total fluctuation of all complexes was the same except for the residual fluctuation of some complexes as seen in Figure 3B. In case of 3-*trans*-*p*-coumaroyl maslinic acid fluctuations were observed in loop region. In general, all systems were stable without fluctuation, and the N-terminal fluctuation was less.

#### 2.4.3. Radius of Gyration (RoG)

The radius of gyration (RG) reveals the density of the system, and ultimately influences the folding rate and stability of proteins. Rg was determined to test the compactness of all complexes and it was found that Rg of all systems was constant with the RMSD of system. This reveals that protein remained stable and compact all through the 20 ns time (Figure 3C). But, Silvestrol and Betulonic acid exhibited less compactness as compare to Reference.

#### 2.4.4. Potential Binding Energy, Hydrogen Bond Analysis and Solvent Accessible Surface Area (SASA)

**Figure 4:**
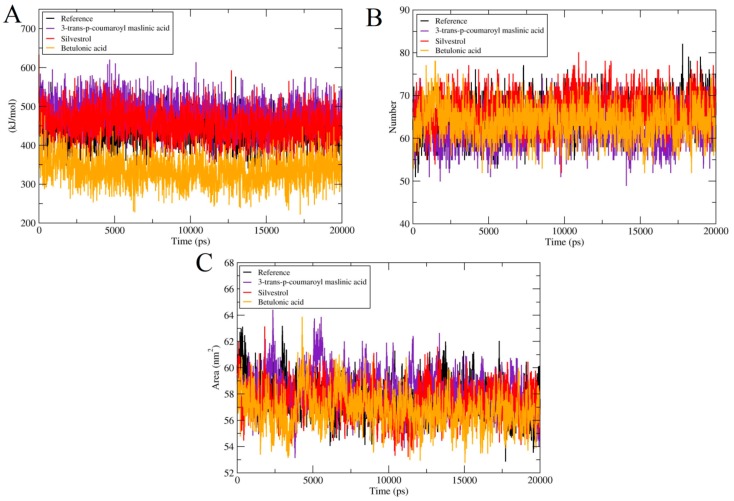
Comparison and detail representation of (**A**) Rg: Radius of gyration, (**B**) hydrogen bond interactions and (**C**) solvent-accessible surface area over the time of 20 ns of Reference and all terpenes complexes.

Potential energies were measured for all docked complexes and Apo structure. All the systems showed steady behavior all over the 20 ns simulations as displayed in Figure 4A. Hydrogen bond is very important for the stability of protein complexes. Hydrogen bonds are a major stabilizing force for the protein and are essential for the structure Hydrogen bond donors/acceptors that do not actually form a hydrogen bond are energetically unfavorable. In folded proteins, on average virtually all possible donors/acceptors make hydrogen bonds-either within the protein or to the solvent (water). As for as Betulonic acid is concerned, only a few hydrogen bonds fluctuations were noticed. In 3-*trans*-*p*-coumaroyl maslinic acid and Silvestrol, a large number of hydrogen bonds were fluctuated. The protein complexes hydrogen bond interaction mode remained stable throughout the simulation process, suggesting that the protein internal hydrogen bonds were stable in all the three cases throughout the 20 ns simulation (Figure 4B). Solvent accessible surface area (SASA) is another key step to maintain protein folding, stability and conformational changes. For all the complexes, SASA analysis was also conducted. The values of SASA for all the systems were ranged from 54–62 nm demonstrating that there were no noteworthy variations in accessibility area of all the systems during the process of simulation. Figure 4C shows the SASA of all systems.

### 2.5. Secondary Structure Analysis

**Figure 5:**
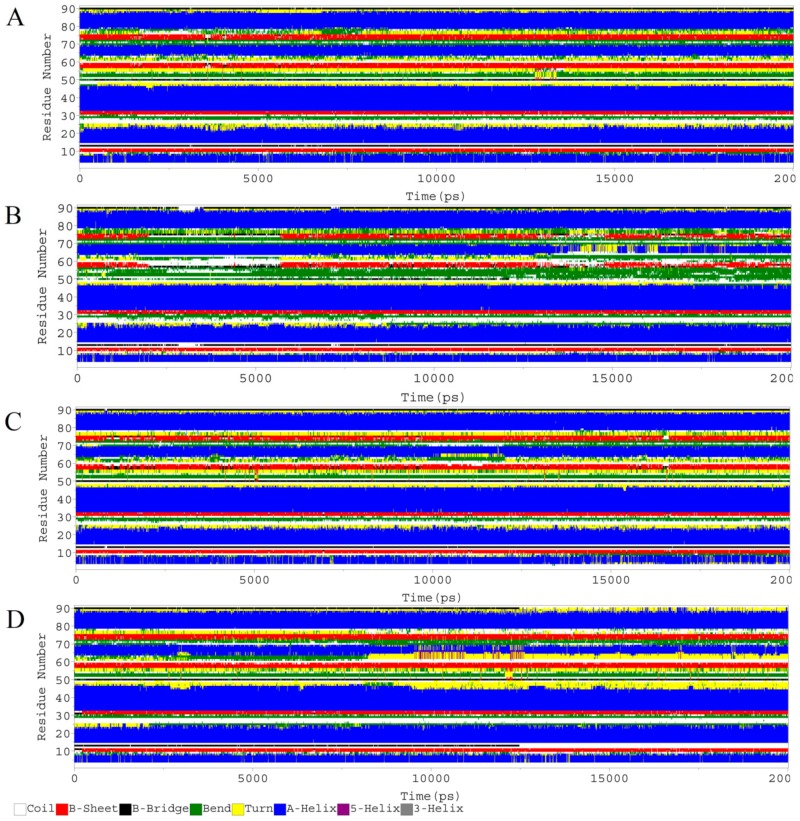
Secondary structure analysis of MDM2 and all the hits over the time of 20 ns. (**A**) Reference, (**B**) 3-*trans*-*p*-coumaroyl maslinic acid, (**C**) Silvestrol and (**D**) Betulonic acid (The residues of MDM2 are shown along Y-axis).

The secondary structures of all complexes were analyzed by DSSP tool in Gramacs. Figure 5 shows the secondary structure plots for all complexes. All the complexes remained structurally conserved. A minute structural transitions was noticed in region (K51-T63) and (H73-S78) in case of 3-*trans*-*p*-coumaroyl maslinic acid and Silvestrol. In addition to some fluctuations in the secondary structure. Generally, the secondary structure remained well-maintained in MDM2 and all the complexes and consistent with thRMSF analysis of all the complexes.

### 2.6. Principle Component Analysis

**Figure 6:**
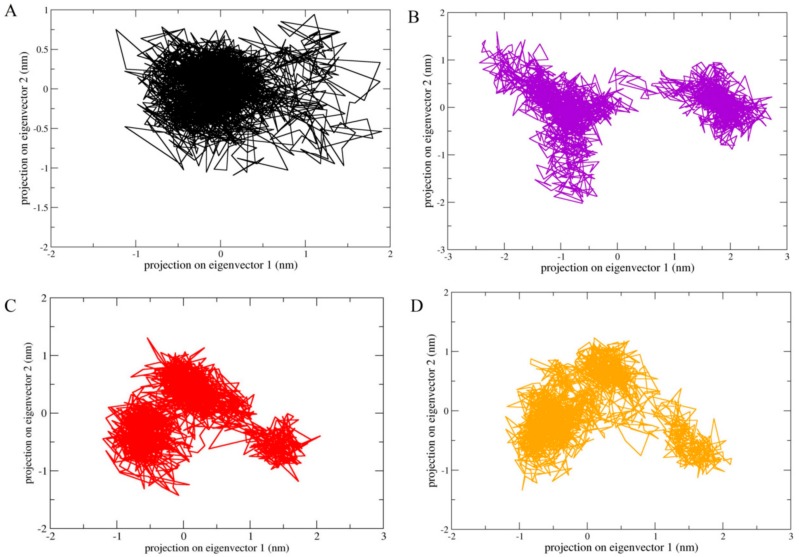
Two-dimensional projection of motion of trajectory of MDM2 bound with hits over the PC1 and PC2. (**A**) Reference, (**B**) 3-*trans*-*p*-coumaroyl maslinic acid, (**C**) Silvestrol and (**D**) Betulonic acid.

The essential dynamic method was used to study the conformational space of MDM2 combined with numerous inhibitory compounds such as 3-*trans*-*p*-coumaroyl maslinic acid, Silvestrol and Betulonic acid. In general, the comparison of the position transitions of ligand bound MDM2 was made with reference. The covariance plot showed the positive and negative limits, which are attributed to the correlated and anti-correlated motions of all protein atoms in the same and opposite directions, respectively. The correlation covariance heat maps for all the complexes are presented in Supplementary Figure S3. The observation suggested that 3-*trans*-*p*-coumaroyl maslinic acid and Betulonic acid covariance plot atomistic changeable data point have similarity with respect to reference, it is aligned with RMSD analysis. The most anti-correlated motion of atoms observed in Silvestrol as compared to reference. In order to further understand the configurational space, we draw the projection of the first principal component and the second principal component, which correspond to the highest position transition in all our systems. Figure 6 shows the PCA plots for complexes and as wee as for reference. The changes of protein movement were observed in all complexes. The results showed that the conformational space of MDM2 inhibitor covered projection increases with the increase of trace value. Therefore, the results of projections show that Betulonic acid has some similarity with reference compared to other two compounds.

## 3. Materials and Methods

### 3.1. Selection and Preparation of Receptor

Downloading of receptor coordinates was done from PDB (PDB ID: 4HG7). Protein structure was prepared using Autodock tools [49,50]. The removal of crystallographic molecules was done before docking. Protein was subjected to Gassteiger partial atomic charges while Autodock tools were used to add polar hydrogen atoms to the protein.

### 3.2. Ligand Database Preparation

Plant bioactive terpenes having antitumor activities were screened through intensive literature search. Different database were used to obtain chemical structures of terpenes such as MPD3 database [26], Pubchem [51], NPACT database [52] and Zinc database [53]. The terpenes molecules were extracted in multiple ligand file formats i.e., sdf, mol, mol2. Moreover, these terpenes structures were optimized in molecular operating environment (MOE) [54] through the addition of partial charges using Protonate 3D module. MMFF94X force field was used for the energy minimization of each terpene. Afterwards, individually selected terpenes molecules were added to the MOE ligand database for the purpose of docking.

### 3.3. Pharmacophore Modeling, Virtual Screening, Drug Likeness Testing and Similarity Searching

Virtual screening is one of the most effective and time-saving methods to discover new, efficient and drug-like compounds for Computer Aided Drug Designing (CADD) [35]. Virtual screening based on pharmacophore was performed on developed test database of terpenes using nutlin-3a bound to MDM2 (PDB ID: 4HG7) as a reference. The MOE pharmacophore module was used for this analysis. The developed pharmacophore system was further analyzed by means of a test database of known inhibitors [35].

Virtual screening based on Tanimoto coefficient was also conducted in order to discover analogous compounds i.e., shape and identical functional groups to the nutlin-3a compound [55]. Compounds having Tanimoto coefficient >0.4 were only chosen for further molecular docking studies.

Lipinski’s five rules were used in order to further evaluate and validate the drug ability of these hit compounds [56]. These rules describe that molecules that fit the criteria of being drug-like should have log *p*-value < 5, hydrogen bond acceptors < 10, molecular weight < 500 Da, and hydrogen bond donors < 5. Deviations from these rules may result in poor penetration or absorption of the compound [56]. A relaxed Lipinski’s rule of five criterion was also applied and only those terpenes hits which were able to pass the criteria were further analyzed.

After the above-mentioned analyses, all compounds were further processed for docking using Auto dock tools. The addition of hydrogen atoms was done while gassteiger partial atomic charges were also calculated.

### 3.4. Rigid Docking

Rigid molecular docking was done by means of Autodock Vina [57]. Number of modes for each ligand was set to 20. The grid box size was (35x*35y*35z) points center on the active site of the MDM2 −26.417, 14.360, −11.915 (x, y, z). Autodock Vina uses empirical scoring function to generate and rank poses. In order to validate the Autodock Vina protocol, we redocked the crystallographic structure of nutlin-3 into the active site of MDM2. For docking experiments, protein was maintained rigid. The compounds after docking were sorted and screened according to docking scores. The docking score of natural ligand (nutlin-3a) is used to set the standard for the second step docking of selected compounds.

### 3.5. Flexible Docking and Clustering

During second stage of docking, compounds were chosen on docking score basis which were again processed for flexible docking by means of Autodock Vina [57]. Residues of binding pocket were kept flexible. 20 confirmations were produced for each compound in contradiction of binding site of MDM2 and the exhaustiveness was maintained to 20. After flexible docking, docking poses were clustered using *K*-means method. Elbow method was used to determine the number of clusters. Docked poses were clustered into three clusters. We picked one compound representative from each cluster for further analyses. Figures were prepared using Pymol [58].

### 3.6. Molecular Dynamics Simulation

Explicit solvent molecular dynamics simulations were done by means of GROMACS 5.1.4 to assess the stability and binding behavior of complex [59]. GROMOS96 43a1 force field [60] was used to get protein topology parameters. For obtaining ligand topology and force field parameters, PRODRG server was applied [61]. Complexes of ligand-protein obtained after docking were solvated in the Cubical box using a model named as simple point charge (SPC) water model. The System neutralization was done by means of replacing some water molecules with CL-ions. To equilibrate the system temperature from 0 to 300 *K* gradually for 50 ps, NVT ensemble was applied. Additionally, system was simulated under NPT ensemble at a pressure and temperature of 1.0 bar 300 *K*, respectively [62]. Particle Mesh Ewald was conducted to calculate the long-range interactions whereas for short-range Van der Waals and Electrostatics, a distance cut off 10A was established [63]. In order to constrain all bonds, LINCS (Linear Constraints Solver) algorithm was applied [64]. Lastly, 20 ns production run was done and trajectories were saved after every 2 fs for each complex. Root mean square deviation (RMSD), Hydrogen bond analysis, Radius of gyration (Rg), Potential Energy, Root mean square fluctuations (RMSF), Secondary structure analyses and SASA [29] were done by means of GROMCAS tools.

### 3.7. Principle Component Analysis

Quasiharmonic technique is a dimension reduction approach dividing conformational space into essential plane and non-essential plane. Essential configurational space enticements the enharmonic degree of freedom related to the dynamic objects [29,65,66]. In PCA approach, Cα atoms average interatomic distance fluctuation was used for covariance matrix. Moreover, set of eigenvalues and analogous eigenvectors were attained subsequently the diagonalization of the covariance matrix. Eigenvectors show the direction of motion in 3D space cooperatively. Then, the principal components (PCS) were managed in downward order with respect to their corresponding eigenvalues. The first two PCs comprised of more than 80% of the total position fluctuation which was assessed by ED analysis for the demonstration of protein’s motion. The movement of protein was proved by ED analysis. The comparative structural changes of all MDM2 ligand binding systems can be recognized by intrinsic dynamics. GROMACS inbuilt modules g_covar & g_anaeig were engaged to perform PCA [67].

## 4. Conclusions

The potency of plant extracted terpenes as antagonists of p53-MDM2 interaction to reactivate p53 functioning was the prime objective of our research. Our study yielded three novel terpenes hits: 3-*trans*-*p*-coumaroyl maslinic acid, Silvestrol and Betulonic acid as prospective antagonists. All the compounds displayed sturdy binding affinity with MDM2 active site compared with that displayed by Nutlin-3a, its co-crystalized ligand. These compounds have a tendency to occupy the regions of p53 binding on MDM2 with inhibitory effect. Therefore, there is a need to explore more about these complexes by conducting in vivo and other analytical techniques. In conclusion, the study offers a strong potential in developing new, cost-effective, and safe plant-based natural drugs against cancer.

## Figures and Tables

**Table 1:** Details of finally selected terpenes hits.

IDs	Terpene	Plant Source	Chemical Structure	Molecular Weight (g/mol)	xLogP	Hydrogen Bond Donor	Hydrogen Bond Acceptor	Chemical Formula	Reference
ZINC ID: ZINC44358756	3-*trans*-*p*-coumaroyl maslinic acid	Ziziphus jujuba	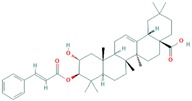	602.8	9.2	2	5	C_39_H_54_O_5_	[37]
NPACT ID: NPACT00946	Silvestrol	Aglaia silvestris	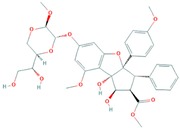	654.7	1.6	4	13	C_34_H_38_O_13_	[38]
PubChem CID: 122844	Betulonic acid	Fructus Jujubae	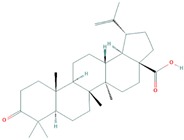	454.7	7.9	1	3	C_30_H_46_O_3_	[39]

**Table 2:** Binding energy score and details of interacting residues of finally selected terpenes hits against MDM2.

Terpene	Binding Energy	Interacting Residue
3-*trans*-*p*-coumaroyl maslinic acid	−22.60	Leu 54, Ile 61, Met 62, Val 75, Phe 86, Phe 91, Val 93, His 96, Ile 99 and Tyr 100
Silvestrol	−20.75	Gln 24, Leu 54, Ile 61, Met 62, Val 75, Phe 91, Val 93, His 96, Ile 99 and Tyr 100
Betulonic acid	−18.83	Leu 54, Ile 61, Met 62, Val 75, Phe 86, Phe 91, Val 93, His 96, Ile 99 and Tyr 100
Reference (Nutlin)	−12.67	Leu 54, Ile 61, Met 62, Val 75, Phe 86, Phe 91, Val 93, His 96, Ile 99 and Tyr 100

## Supplementary Materials

The following are available online, Figure S1: A schematic flow chart of methodology used in this study, Figure S2: 2D Binding interactions of terpenes with active site residues of MDM2-P53 (A) 3-*trans*-*p*-coumaroyl maslinic acid, (B) Silvestrol and (C) Betulonic acid, Figure S3: Covariance matrices of all the complexes during 20ns Simulations: (A) Reference, (B) 3-*trans*-*p*-coumaroyl maslinic acid, (C) Silvestrol and (D) Betulonic acid.
