# Supplementary material for: Promising Terpenes as Natural Antagonists of Cancer: An In-Silico Approach
Source: Molecules. 2019 Dec 30;25(1):155. doi: 10.3390/molecules25010155 (PMC6983034; doi:10.3390/molecules25010155)
Supplement: Supplementary file 1 [file molecules-25-00155-s001.pdf]

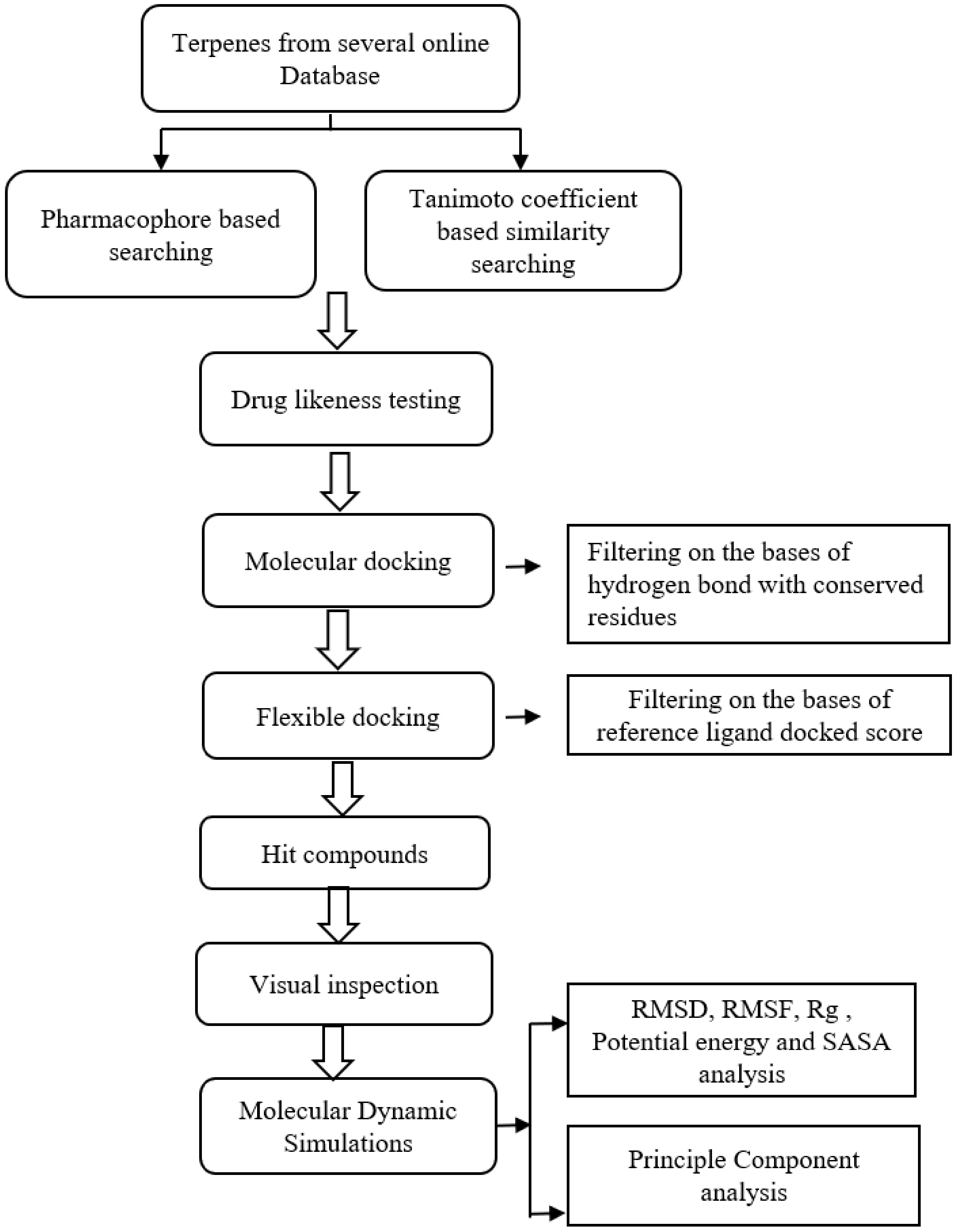

Supplementary figure 1. A schematic flow chart of methodology used in this study.

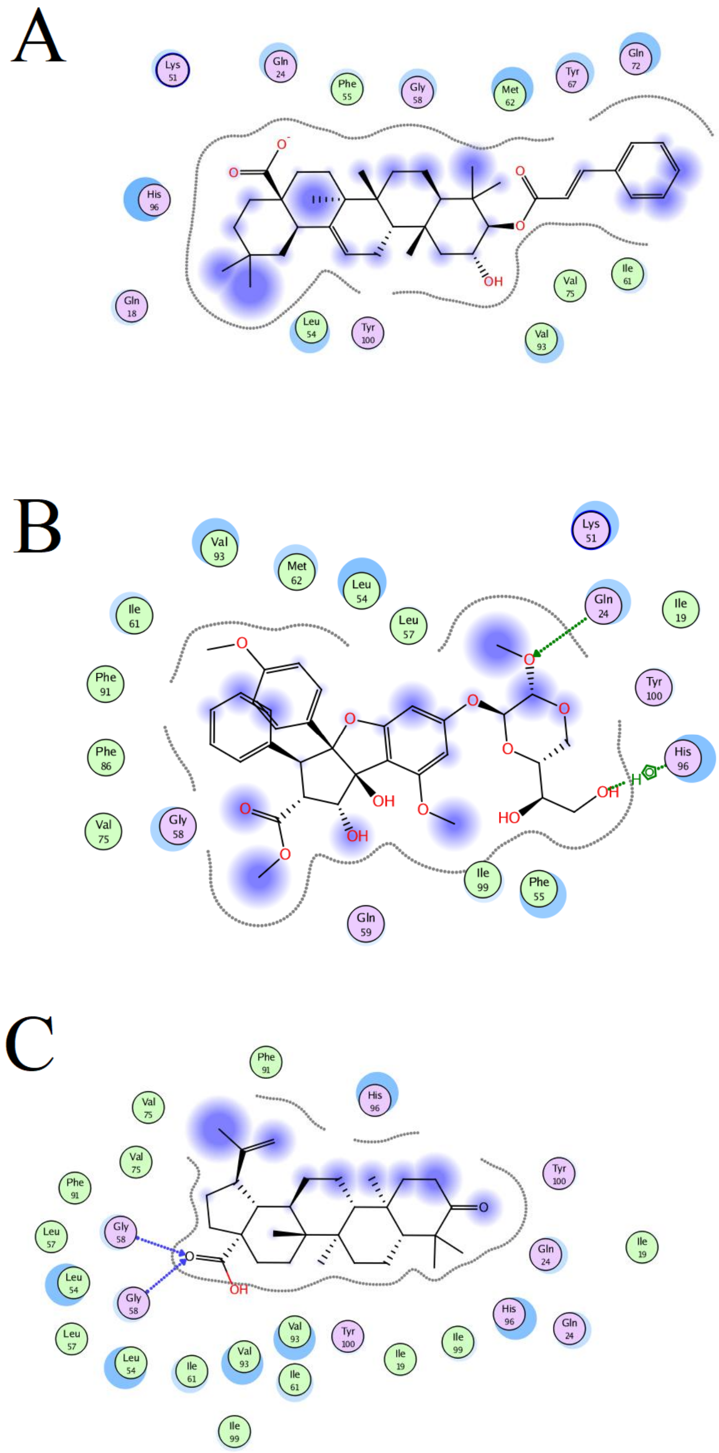

Supplementary figure 2. 2D Binding interactions of terpenes with active site residues of MDM2-P53 (A) 3-trans-p-coumaroyl maslinic acid, (B) Silvestrol and (C) Betulonic acid

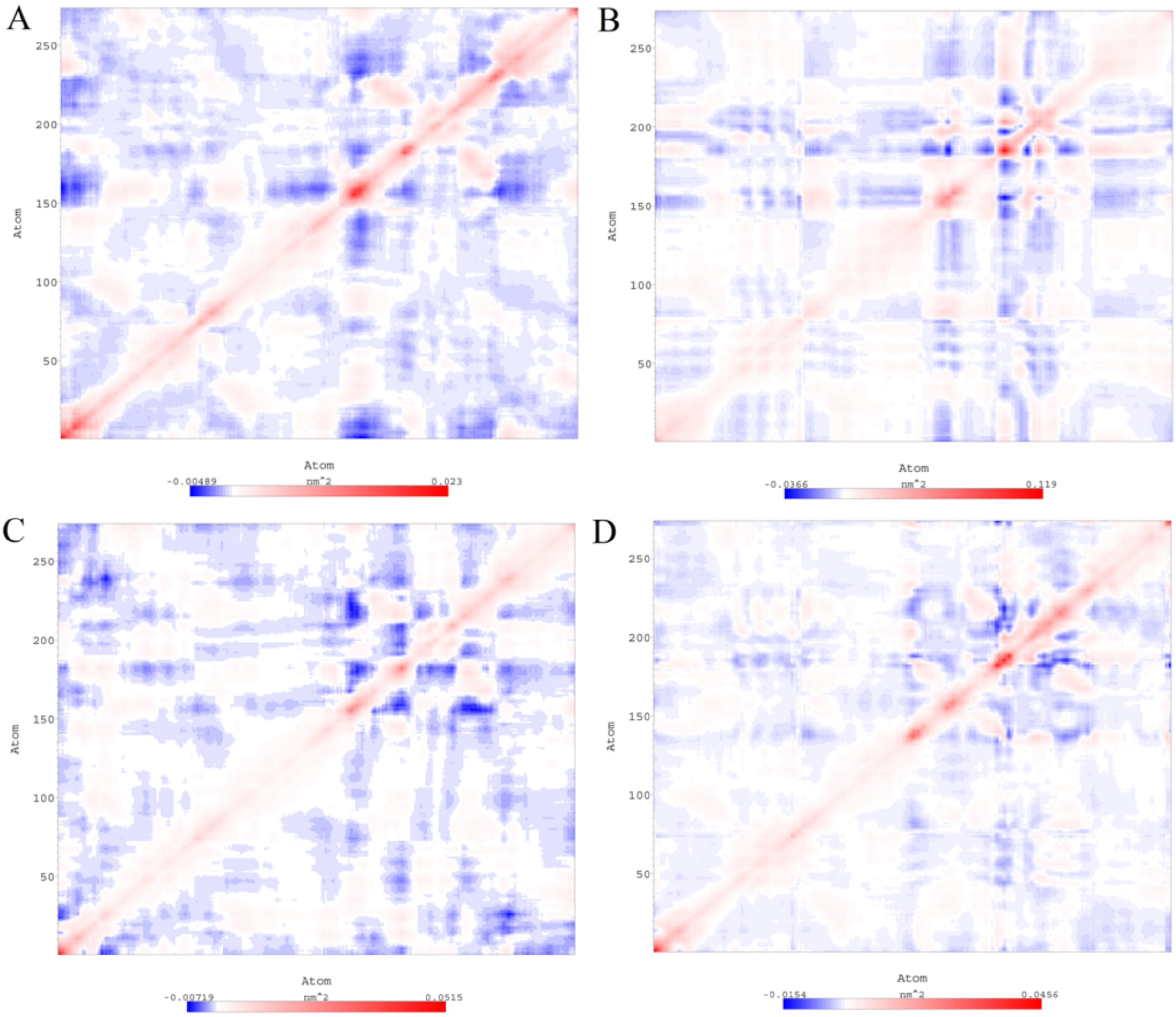

Supplementary figure 3. Covariance matrices of all the complexes during 20 ns Simulations: (A) Reference, (B) 3-trans-p-coumaroyl maslinic acid, (C) Silvestrol and (D) Betulonic acid
